# Developing a Situational Judgement Test to Assess Clinical Judgement in Fourth-Year Medical Students: A Pilot Study

**DOI:** 10.7759/cureus.66530

**Published:** 2024-08-09

**Authors:** Kyle M Rei, Maegen Dupper, Vy Han, Rajuno Ettarh

**Affiliations:** 1 Medical Education, California University of Science and Medicine, Colton, USA

**Keywords:** situational judgement test, objective structured clinical exam (osce), validation, clinical judgement, medical education

## Abstract

Introduction

Assessing clinical judgement objectively and economically presents a challenge in academic medicine. The authors developed a situational judgement test (SJT) to measure fourth-year medical students’ clinical judgement.

Methods

A knowledge-based, single-best-answer SJT was developed by a panel of subject matter experts (SMEs). The SJT included 30 scenarios, each with five response options ranked ordinally from most to least appropriate. A computer-based format was used, and the SJT was piloted by two cohorts of fourth-year medical students at California University of Science and Medicine in 2022 and 2023 upon completion of an internship preparation course. Subsequently, students completed an optional survey. Evaluated scoring methods included original ordinal ranking, dichotomous, dichotomous with negative correction, distance from SME best answer, and distance from SME best answer squared.

Results

The SJT was completed by 142 fourth-year medical students. Cronbach’s alpha ranged from 0.39 to 0.85, depending on the scoring method used. The distance-from-SME-best-answer-squared method yielded the highest internal consistency, which was considered acceptable. Using this scoring method, the mean score was 72.89 (SD = 48.32, range = 26-417), and the standard error of measurement was 18.41. Item analysis found that seven (23%) scenarios were of average difficulty, 13 (43%) had a good or satisfactory discrimination index, and nine (30%) had a distractor efficiency of at least 66%. Most students preferred the SJT to a traditional multiple-choice exam (16; 62%) and felt it was an appropriate tool to assess clinical judgement (15; 58%).

Conclusions

The authors developed and piloted an SJT to assess clinical judgement among medical students. Although not achieving validation, subsequent development of the SJT will focus on expanding the SME concordance panel, improving difficulty and discrimination indices, and conducting parallel forms reliability and adverse impact analyses.

## Introduction

While some medical inquiries have been answered with data at the highest level of evidence hierarchies, other inquiries remain more elusive and present a gray area in medicine [1]. The role of the physician is to contend with this uncertainty by using clinical judgement, which extends beyond theoretical knowledge and incorporates elements such as personal experience, patient perspectives, and other insights [2]. Developed through practice, experience, knowledge, and continuous critical analysis, clinical judgement is a teachable attribute [3]. However, assessing clinical judgement objectively and economically presents a challenge in academic medicine [4].

While the traditional objective structured clinical examination (OSCE) is a widely used methodology to assess clinical competencies, common concerns about this testing format include its high demand for resources, scalability for larger cohorts, and feasibility for assessing clinical judgement in scenarios that may not have only one correct answer [4-6]. Compared to the traditional OSCE, the situational judgement test (SJT) demands significantly fewer resources as it can be computer-delivered and machine-marked while still facilitating the assessment of traits transcending pure clinical knowledge [7].

SJTs commonly present examinees with a variety of role-relevant scenarios developed by subject matter experts (SMEs) and task examinees to identify the appropriateness of different responses to each scenario [7]. However, the SJT is more of a measurement methodology than a single style of test and has wide variability in construction and application [7]. Notably, in addition to its ability to predict performance with respect to domain-based knowledge, SJT also reliably measures nonacademic constructs and interpersonal skills [8]. While the SJT has been used primarily for personnel selection in the last half century, we propose using this measurement methodology to assess clinical judgement among medical students [9].

The primary objectives of this study were to develop and validate an SJT to assess clinical judgement among fourth-year medical students following the completion of an internship preparation course, evaluate the psychometric properties of the SJT, determine the reliability of different SJT scoring methods, assess the acceptability and perceived effectiveness of the SJT among medical students, and provide recommendations for future use and improvement of the SJT.

## Materials and methods

SJT construction

An SJT was constructed to assess the clinical judgement of fourth-year medical students at California University of Science and Medicine (CUSM), Colton, United States, in 2022 and 2023 after completing an internship preparation course. The SJT was developed according to the guidelines presented by Patterson et al. [7]. A panel of two SMEs constructed 30 scenarios likely to be encountered as an intern, along with five response options for each scenario. SMEs collaboratively developed a scoring key that assigned an ordinal ranking to each response based on appropriateness. The response instructions were knowledge-based (i.e., what is the best option), and the response format of single best response was chosen for simplicity: examinees selected only one out of the five responses for each scenario as reflecting the best clinical judgement. The SJT was then constructed in a computer-based format using ExamSoft software (ExamSoft Worldwide LLC, Oakland, United States) and was piloted by two sequential cohorts of students.

Scoring methods

All statistics were performed using Microsoft Excel Version 2406 (Microsoft Corporation, Redmond, United States). Several data transformations were performed to empirically evaluate the effects of different scoring methods. The original data included scores per scenario ranging from 1 to 5, with 5 representing the most appropriate response. The first data transformation created dichotomous outcomes by defining a correct response as either of the best two responses, while an incorrect response was defined as any other response. The second data transformation was dichotomous with a negative correction, which deducted a point for incorrect responses. The third data transformation was the distance from the SME’s best answer, which subtracted the original scenario score from 5 and defined zero as the best score. The final data transformation was the distance from the SME’s best answer squared.

Validity

Content validity is the extent to which the scenarios assess the trait that the exam is designed to measure [10]. SMEs developed the scenarios to assess clinical judgement using the following criteria: the scenario is likely to be encountered in clinical medicine, a single objectively correct response based solely upon clinical knowledge does not exist, and the scenario demonstrates a subset of themes identified by the Association of American Medical Colleges’ premed professional competencies, including cultural awareness and humility, empathy and compassion, ethical responsibility, interpersonal skills, and resilience and adaptability [11].

Reliability

Internal consistency describes the degree to which all the items in an exam measure the same concept (e.g., clinical judgement) and therefore also reflects the interrelatedness of the items within the exam [12]. Internal consistency was measured via Cronbach’s alpha for each scoring method, which was calculated using Equation 1. Criteria for alpha were as follows: α < 0.6: unreliable; α = 0.6-0.7: marginally reliable; and α > 0.7: relatively reliable [10]. An additional measure of reliability is the standard error of measurement (SEM), which describes the likely range of true scores an examinee may achieve due to the unreliability of the assessment [13]. SEM was also found for each scoring method, which was calculated using Equation 2.

Equation 1: Cronbach’s alpha

$\alpha\ =\ \frac{k}{k-1}\left(1-\frac{\sum\sigma_k^2}{\sigma_{Total}^2}\right)$,

where α = Cronbach’s alpha and k = the number of items.

Equation 2: SEM

$SEM=SD\sqrt{1-\alpha}$,

where SEM = standard error of measurement, SD = standard deviation, and α = Cronbach’s alpha.

Item analysis

Scenario quality was measured with the difficulty index (p-value), discrimination index (DI), and distractor efficiency (DE) using dichotomized data [14]. High-scoring and low-scoring groups of examinees were identified using the upper and lower 27th percentiles of students, respectively. p-Values reflect the difficulty of each scenario and were calculated using Equation 3, where H and L represent the number of correct responses from high- and low-scoring groups, and N represents the total number of students in both groups. Criteria for p-values were as follows: p > 70: easy; p = 30-70: average; and p < 30: difficult [14]. DI reflects the ability of a question to discriminate between high- and low-scoring students, such that a value of zero indicates that an equal number of high and low-scoring students correctly answered a question. DI was calculated using Equation 4, maintaining the same variable definitions as above. Criteria for DI values were as follows: DI >0.25 is good, DI = 0.24-0.17 is satisfactory, DI = 0.16-0.13 is moderate, and DI <0.13 is limited [15]. Lastly, DE reflects the proportion of non-functional distractors (NF-Ds), which are incorrect response options for a scenario selected by less than 5% of examinees. DE was calculated as follows: 3 NF-D: DE = 0%; 2 NF-D: DE = 33%; 1 NF-D: DE = 66%; and no NF-D: DE = 100% [14].

Equation 3: Difficulty index

$p=100\left(\frac{H+L}{N}\right)$,

where p = difficulty index, H = number of high group (73rd% tile) correct responses, L = number of low group (23rd% tile) correct responses, and N = total number of students in both high and low groups.

Equation 4: DI

$DI=2\left(\frac{H-L}{N}\right)$,

where DI = discrimination index, H = number of high group (73rd% tile) correct responses, L = number of low group (23rd% tile) correct responses, and N = total number of students in both high and low groups.

Survey

The 2022 cohort was administered an optional four-item dichotomous survey to gauge examinee reactions to the SJT immediately following their completion of the test. The survey evaluated whether the SJT assessed clinical judgement as it was delivered in the course, was preferable to a traditional multiple-choice exam, was an appropriate tool to assess clinical judgement, and included common clinical scenarios. The survey also included an optional free-response comment. Dichotomous responses were summarized graphically, and free responses were analyzed according to theme.

IRB approval

Exempt approval was obtained for protocol HS-2022-06 from the IRB in connection with the CUSM.

## Results

The SJT was administered to 142 fourth-year medical students across two consecutive cohorts after completing an internship preparation course. The first cohort included 60 students, and the second cohort included 82 students. The psychometric analysis included item analysis in the form of difficulty index (p-value), DI, DE, and reliability in the form of internal consistency (alpha) and SEM. Pooled results (n = 142) are displayed in Table 1.

**Table 1:** SJT item analysis output ^1 ^The p-value reflects the difficulty index and should not be confused with statistical significance. Item analysis used dichotomized data, with the top two responses as indicated by SMEs defining the correct response. DI: discrimination index; DE: distractor efficiency; p-value: difficulty index; SJT: situational judgement test; SME: subject matter expert

Scenario	Response 5 (best)	Response 4	Response 3	Response 2	Response 1 (worst)	No response	p-Value^1^	DI	DE
1	93%	6%	1%	0%	0%	0%	98	0	0%
2	0%	7%	91%	1%	1%	0%	6	-0.09	33%
3	66%	6%	6%	1%	13%	8%	70	0.29	66%
4	15%	5%	4%	49%	24%	2%	23	0.4	66%
5	8%	17%	5%	67%	1%	2%	26	0.23	33%
6	6%	63%	25%	1%	1%	2%	71	0.31	33%
7	79%	2%	10%	6%	1%	2%	79	0.34	66%
8	63%	27%	9%	0%	0%	1%	91	0.03	33%
9	26%	73%	0%	0%	0%	1%	98	0.06	0%
10	30%	16%	17%	36%	0%	1%	43	0.37	66%
11	18%	50%	11%	20%	0%	1%	68	0.37	66%
12	11%	75%	14%	1%	0%	0%	86	0.23	33%
13	13%	13%	25%	47%	0%	2%	28	0.37	66%
14	42%	23%	27%	7%	0%	1%	63	0.29	66%
15	62%	25%	8%	1%	4%	0%	87	0.11	33%
16	94%	3%	4%	0%	0%	0%	97	0.03	0%
17	62%	27%	1%	9%	0%	1%	89	0.17	33%
18	9%	25%	24%	42%	0%	1%	34	0.49	66%
19	21%	75%	1%	3%	0%	0%	96	0.09	0%
20	63%	9%	1%	25%	0%	1%	70	0.26	33%
21	79%	19%	0%	1%	1%	0%	98	0.03	0%
22	44%	48%	1%	6%	0%	1%	91	0.14	33%
23	30%	69%	0%	0%	0%	1%	99	0.03	0%
24	77%	15%	2%	5%	0%	1%	95	0.14	0%
25	58%	9%	20%	11%	0%	2%	65	0.54	66%
26	75%	11%	4%	7%	0%	3%	86	0.26	33%
27	1%	26%	68%	4%	0%	1%	28	0.26	33%
28	34%	46%	19%	0%	0%	1%	80	0.11	33%
29	87%	7%	2%	0%	1%	2%	95	0.14	0%
30	97%	1%	1%	1%	0%	0%	97	-0.03	0%

Item analysis

**Figure 1:**
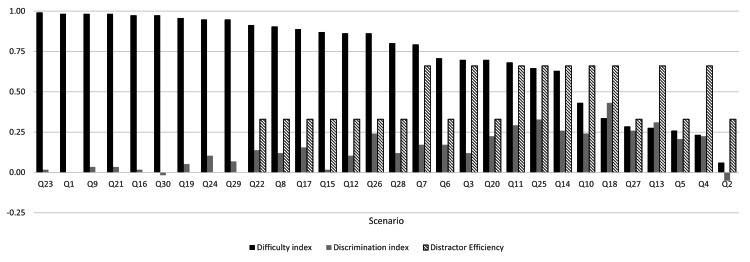
SJT item analysis plot including difficulty index, DI, and DE Scenarios are arranged from left to right in descending order of difficulty index. The difficulty index is presented as p-value/100 to share the axis with the other two factors of item analysis. All factors of item analysis were found using dichotomized data. DE: distractor efficiency; DI: discrimination index; SJT: situational judgement test

Item analysis results are displayed graphically in Figure 1 and summarized in Table 2 according to criteria classification. Difficulty index analysis found that among the 30 scenarios, 18 (60%) were classified as easy (p > 70), seven (23%) were average (p = 30-70), and five (17%) were difficult (p < 30). DI analysis found that six (20%) scenarios had good discrimination between high- vs. low-scoring groups (DI >0.25), seven (23%) had satisfactory discrimination (DI = 0.17-0.25), two (7%) had moderate discrimination (DI = 0.13-0.16), and 15 (50%) had limited discrimination (DI <0.13). DE analysis found that no scenarios had a DE of 100%, nine (30%) scenarios had a DE of 66%, 12 (40%) scenarios had a DE of 33%, and nine (30%) scenarios had a DE of 0%. Using both p-value and DI as indicators of scenario quality, six (20%) scenarios had an average difficulty with good or satisfactory discrimination.

**Table 2:** SJT item analysis classification of difficulty index, DI, and DE All factors of item analysis were found using dichotomized data. DE: distractor efficiency; DI: discrimination index; NF-D: non-functioning distractors; SJT: situational judgement test

Criteria	Description	Scenarios, N (%)
Difficulty index		
>70	Easy	18 (60%)
30-70	Average	7 (23%)
<30	Difficult	5 (17%)
DI		
>0.25	Good	6 (20%)
0.17-0.25	Satisfactory	7 (23%)
0.13-0.16	Moderate	2 (7%)
<0.13	Limited	15 (50%)
DE		
0 NF-D	DE = 100%	0 (0%)
1 NF-D	DE = 66%	9 (30%)
2 NF-D	DE = 33%	12 (40%)
3 NF-D	DE = 0%	9 (30%)

Reliability

Reliability results are summarized in Table 3 and include internal consistency reported as Cronbach’s alpha and SEM for each scoring method. Depending on the scoring method used, alpha ranged from 0.39 (α < 0.6: unreliable) in the dichotomous and dichotomous-with-negative-correction methods to 0.85 (α > 0.7: relatively reliable) in the distance-from-SME-best-answer-squared method. Related to this finding, SD ranged from 2.46 in the dichotomous method to 48.32 in the distance-from-SME-best-answer-squared method. Comparing dichotomous vs. dichotomous-with-negative-correction, they shared an alpha of 0.39. However, adding a negative correction increased the SD from 2.46 to 4.91, which increased the SEM from 1.92 to 3.83.

**Table 3:** SJT scoring method, descriptive statistics, Cronbach’s alpha, and SEM Dichotomized data defined the top two responses, as indicated by SMEs, as the correct response. A negative correction deducted one point for an incorrect response. The distance-from-SME-best-answer method subtracted the original response score from 5, with 0 indicating the best response. SEM values are presented in units of exam points, not percentages. SEM: standard error of measurement; SJT: situational judgement test; SME: subject matter expert

Scoring method	Mean	SD	Range	Theoretical range	Alpha	SEM
Original data	119.65	9.63	57-134	0-150	0.73	5.03
Dichotomous	21.63	2.46	12-27	0-30	0.39	1.92
Dichotomous with a negative correction	13.27	4.91	-6-24	-30-30	0.39	3.83
Distance from the SME’s best answer	30.35	9.63	16-93	0-150	0.73	5.03
Distance from SME’s best answer squared	72.89	48.32	26-417	0-750	0.85	18.41

Cohort comparison

Item analysis using the original data scoring method yielded similar results in both cohorts with respect to difficulty index, DI, and DE. However, reliability analysis was remarkable for the alpha of 0.84 vs. 0.19 and SEM of 5.35 vs. 4.73 when comparing the first and second cohorts, respectively.

Survey

**Figure 2:**
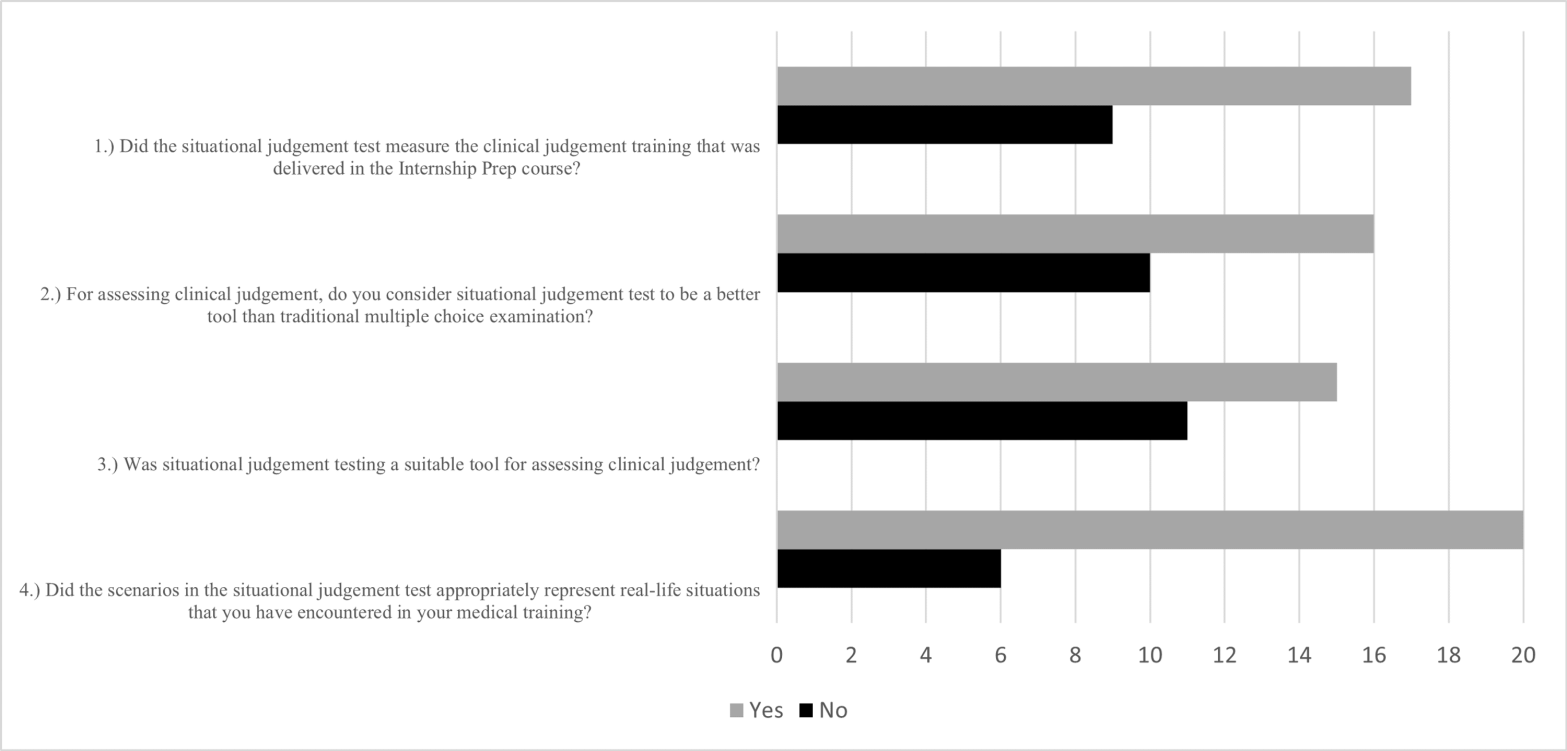
SJT survey responses An optional four-item dichotomous survey was administered to the first cohort. Out of 60 examinees invited to participate, 26 completed the survey. SJT: situational judgement test

Out of the 60 first-cohort examinees invited to participate, 26 (43%) completed the survey (Figure 2). The survey found that 17 (65%) respondents agreed that the SJT assessed clinical judgement as it was delivered in the course, 16 (62%) respondents agreed that the SJT was preferable to a traditional multiple-choice exam, 15 (58%) respondents agreed that the SJT was an appropriate tool to assess clinical judgement, and 20 (77%) respondents agreed that the scenarios included common clinical scenarios. Six free-response comments were received and shared the common theme that examinees experienced discomfort choosing between multiple responses with at least moderate levels of appropriateness.

## Discussion

Reliability and scoring method

While SJTs have been used for assessing interpersonal skills in medical school admission, an SJT was created to measure clinical judgement development following the completion of an internship preparation course [16]. Because SJT scenarios are constructed heterogeneously, and therefore evaluate multiple performance dimensions, there is an inherent challenge in measuring the internal consistency of an SJT [17,18]. Cronbach’s alpha was found to vary between cohorts (0.19-0.84) and scoring methods (0.39-0.85). The former finding is explained by the fact that reliability is a characteristic of test scores rather than the test itself [19]. Equation 3 shows that Cronbach’s alpha depends on the total score variance, which will inherently differ between samples of students. Studies investigating this effect argued that heterogeneity between cohorts of examinees could explain differences in measured internal consistency on a single scale [20]. While this phenomenon does not limit the generalizability of validated SJTs, it highlights the need for large samples to validate SJTs. Therefore, for the purpose of validating the SJT, pooled cohort data was used.

The latter finding has been eloquently demonstrated in a study that showed how the scoring method alone could vary the internal consistency of a previously validated SJT between 0.33 and 0.76, which is a range similar to that found in the present study [21]. This variation in alpha is likely explained by the effect that the scoring method can have on total score variance and therefore alpha [21].

Although not selected on an empirical basis alone, the scoring method yielding the highest reliability was distance-from-SME-best-answer-squared. Dichotomizing data adds leniency with respect to selecting inferior responses compared to a traditional multiple-choice exam; however, doing so treats a moderate response equally to the least appropriate response. Using the distance-from-SME-best-answer-squared method accounts for this nuance while also creating greater separation between students through the magnification of errors [21].

Adverse impact

The SJT response instructions were knowledge-based (i.e., what is the best option), which has demonstrated higher criterion-related validity than tests with behavioral tendency instructions (i.e., what would you be most likely to do) [22]. However, a meta-analysis found that knowledge-response instructions showed greater race differences than behavioral tendency instructions [23]. Whetzel et al. Investigated the effects of cognitive load and personality on SJTs and concluded that SJTs are likely to exhibit adverse impacts with respect to race and, to a lesser extent, sex [23]. Smith et al. developed a similar SJT designed to assess professionalism among pharmacy students and performed an adverse impact analysis with findings mirroring those of the meta-analysis: their SJT significantly favored female and white students with small effect sizes [24].

Survey

Survey results revealed that the SJT was generally well received by examinees. While some examinees reported experiencing discomfort choosing between multiple responses with at least moderate levels of appropriateness, most students preferred the SJT to a traditional multiple-choice exam and felt that it was a suitable tool for assessing clinical judgement. This finding supports the face validity of the SJT, which, although independent from more objective metrics for determining validation, should be considered in the construction of an SJT because it influences test-taking motivation [7]. Smith et al. found that pharmacy students perceived their SJT as an opportunity to gain exposure to challenging situations they may later encounter, and the authors concluded that although not their primary research aim, implementing the SJT early in the program may provide useful formative assessment to track development as students progress [24].

Limitations

Content validity could be improved with the use of a larger SME concordance panel, and predictive validity could be assessed longitudinally, similar to Cousans et al., who compared post-graduate supervisor ratings of in-role performance and incidence of remedial action among high vs. low scorers on an SJT [25]. While internal consistency was found to be acceptable in the present study, McDaniel et al. argued that because most SJTs are inherently heterogeneous, a more reasonable estimate of reliability would be derived from test-retest or parallel forms reliability analysis [22]. Item quality analysis highlighted the need for developing more difficult scenarios to better discriminate between high- and low-scoring examinees and improve DE. Demographic data will be collected in future administrations to enable adverse impact analysis via regression analysis. Finally, upon subsequent validation, further studies plan to employ the assessment before and after completion of an internship preparation course to quantify gains in clinical judgement. However, earlier implementation with a longitudinal approach as suggested by Smith et al. will also be considered [24].

## Conclusions

Assessing clinical judgement objectively and economically presents a challenge in academic medicine. An SJT was developed and piloted to assess clinical judgement among fourth-year medical students. Various scoring methods were evaluated, and the distance-from-SME-best-answer-squared method yielded the highest internal consistency, which was considered acceptable, and created the greatest separation between students. Although not achieving validation, subsequent development of the SJT will focus on expanding the SME concordance panel, improving difficulty and discrimination indices, and conducting parallel forms reliability and adverse impact analyses.
